# Prevalence of migalastat-amenable variants in patients with Fabry disease from Brazil, Argentina, and Colombia: an observational study

**DOI:** 10.3389/fgene.2026.1876053

**Published:** 2026-10-05

**Authors:** Juan Manuel Politei, Ana María Zarante Bahamón, Ana Maria Martins, Afrânio Souza De Melo, Marcelo Horacio de Sa Pereira, Andréia Loures-Vale, Biliana O. Veleva-Rotse, Eva Krusinska, Valerie Lengoc

**Affiliations:** 1 Neurology Department, SPINE Fundation, Buenos Aires, Argentina; 2 San Ignacio University Hospital, Bogotá, Colombia; 3 Federal University of São Paulo, São Paulo, Brazil; 4 State University of Rio de Janeiro, Rio de Janeiro, Brazil; 5 Amicus Therapeutics UK Ltd., Marlow, United Kingdom; 6 BioMarin Pharmaceutical Inc., Novato, CA, United States; 7 Amicus Therapeutics Inc., Princeton, NJ, United States; 8 BioMarin UK Ltd., London, United Kingdom

**Keywords:** amenable *GLA* variant, Argentina, Brazil, Colombia, Fabry disease, migalastat, prevalence, South America

## Abstract

**Introduction:**

Fabry disease is a progressive, X-linked lysosomal disorder caused by *GLA* variants that result in dysfunctional α-galactosidase A (α-Gal A) enzyme. One treatment option is migalastat, an oral small-molecule pharmacological chaperone treatment for patients with migalastat-amenable *GLA* variants.

**Methods:**

In this retrospective study, medical records were used to determine the prevalence of migalastat-amenable variants among individuals managed for Fabry disease at centers in Brazil, Argentina, and Colombia (primary endpoint). Demographic and clinical characteristics were explored using available data. Medical records of males or females of any age with a diagnosis of Fabry disease (based on genetic testing and/or α-Gal A enzymatic activity) were eligible for inclusion.

**Results:**

Data were collected for 1,196 individuals diagnosed with Fabry disease in Brazil (n = 1,000), Argentina (n = 122), and Colombia (n = 74); of these, 720 (60.2%) had migalastat-amenable *GLA* variants (650 [65.0%] patients in Brazil, 32 [26.2%] in Argentina, and 38 [51.4%] in Colombia). The migalastat-amenable variants differed by country with little overlap among the cohorts in Brazil, Argentina, and Colombia in the most common variants. Median age (in years) at the study reference date (July 1, 2021) for individuals with migalastat-amenable variants was 45.6, 44.1, and 21.8 years in Brazil, Argentina, and Colombia, respectively. Data on clinical characteristics were available only in Argentina and Colombia.

**Discussion:**

In those for whom data were available, there appeared to be both objective and subjective manifestations of Fabry disease, as evidenced by renal impairment, cardiac involvement, and classic Fabry disease signs and symptoms.

## 1 Introduction

Fabry disease is a progressive, X-linked lysosomal disorder caused by *GLA* variants that result in dysfunctional α-galactosidase A (α-Gal A) enzyme (absent or deficient α-Gal A activity) (Germain, 2010; Ortiz et al., 2018). This causes cellular dysfunction and the accumulation of glycosphingolipids throughout the body, leading to progressive multiorgan dysfunction and morbidity (Schiffmann et al., 2009; Germain, 2010). The clinical presentation and age of onset of Fabry disease are varied; early-onset (classic) Fabry disease presents in childhood, while the later-onset phenotype typically presents in mid-life and is more heterogeneous (Arends et al., 2017; Ortiz et al., 2018; Caputo et al., 2021; Germain et al., 2022). Both male and female individuals with pathogenic *GLA* variants can experience a wide range of symptoms, including neurological, renal, cardiac, and gastrointestinal symptoms (Mehta et al., 2009; Schiffmann et al., 2009; Germain, 2010; Mehta et al., 2010; Ortiz et al., 2018). Renal failure, cardiovascular disease, and cerebrovascular complications are common causes of premature death in patients with Fabry disease (MacDermot et al., 2001; Mehta et al., 2009; Schiffmann et al., 2009; Ortiz et al., 2018).

Treatment options for Fabry disease include intravenous enzyme replacement therapy with agalsidase alfa, agalsidase beta, or pegunigalsidase alfa; and migalastat, an orally administered small-molecule chaperone treatment for patients with Fabry disease who have migalastat-amenable *GLA* variants (PINT-PHARMA International S.A. under license from Amicus Therapeutics UK Ltd, 2025; Bichet et al., 2023; Linhart et al., 2023; Giugliani et al., 2025; Pint Pharma Produtos Médico-Hospitalares e Farmacêuticos Ltda, 2025; Pint Pharma, 2023). Generally, identifying disease-causing genes and variants is important for understanding disease etiology and informing clinical approaches to disease prevention and treatment (He et al., 2022). This is relevant in Fabry disease because characterization of *GLA* variants can support diagnosis and clinical management options. To date, there are only limited data from Latin American countries on the prevalence of migalastat-amenable variants in patients with Fabry disease. The aim of the PRevalence of Fabry dIsease aMEnable mutations to migalastat (PRIME) study was to determine the prevalence of migalastat-amenable variants among individuals managed for Fabry disease at centers in Brazil, Argentina, and Colombia, and to explore the demographic and clinical characteristics of these individuals.

## 2 Methods

Data were retrospectively extracted and anonymized from the medical records of individuals diagnosed with Fabry disease at study centers in Brazil, Argentina, and Colombia. These included index patients at dialysis clinics and related individuals identified by family cascade screening in Brazil, and patients at the principal investigators’ clinics and satellite clinics in Argentina and Colombia. Investigators or other designated study center staff manually reviewed medical records and collected information that was provided at the first center visit (or at the time of Fabry disease diagnosis). Individuals (male or female and of any age) were eligible for inclusion in the study if they had an established diagnosis of Fabry disease based on genetic testing and/or α-Gal A enzymatic activity, and if their medical records contained all key demographic characteristics and outcomes of clinical assessments pertaining to the study endpoints. To make full use of the information that could be collated, however, analyses of demographic and clinical characteristics in this report were undertaken based on available data. Because data were collected retrospectively from medical records, diagnostic approaches, clinical assessments, and variant interpretation reflected local practice at each center and were not standardized across countries.

The studies involving humans were approved by Universidade Federal de São Paulo, São Paulo, Brazil; Iniciativa y Reflexión Bioética, Buenos Aires, Argentina; Comité de Investigaciones y Etica Institucional, Hospital Universitario San Ignacio, Bogota, Colombia. The studies were conducted in accordance with local legislation and institutional requirements. The participants or participants’ legal guardians/next of kin provided their written informed consent to participate in this study.

The primary endpoint was the proportion of Fabry disease patients with variants amenable to migalastat in Brazil, Argentina, and Colombia. Secondary endpoints included describing demographic and clinical characteristics of the amenable population. Individuals’ ages for summaries of demographic or clinical characteristics were calculated against a reference date, nominally determined as July 1, 2021. For an individual’s data to be included in analyses of clinical characteristics restricted to adults (estimated glomerular filtration rate [eGFR] and cardiac parameters), the date on which an assessment (renal or cardiac) was performed had to be documented in their medical record and the individual had to be aged ≥18 years on that date. Descriptive statistical analyses were performed. Categorical variables were summarized using counts and percentages, and continuous variables were summarized using mean, standard deviation, median, and interquartile range. Exact binomial 95% confidence intervals (CIs) were calculated for the prevalence of migalastat-amenable variants overall and by country. No formal statistical comparisons between countries were performed because of differences in cohort ascertainment, diagnostic approaches, and availability of clinical data. Missing data were not imputed; analyses of demographic and clinical characteristics were based on available data. Statistical analyses were performed using SAS® version 9.4 (SAS Institute, Cary, NC, United States).

## 3 Results

### 3.1 Prevalence of migalastat-amenable variants

Data were collected for 1,196 individuals with Fabry disease (Brazil, n = 1,000; Argentina, n = 122; Colombia, n = 74). The overall prevalence of individuals carrying migalastat-amenable variants was 60.2% (720/1,196; exact 95% CI 57.4–63.0), with country-specific estimates of 65.0% in Brazil (650/1,000; exact 95% CI 62.0–68.0), 26.2% in Argentina (32/122; exact 95% CI 18.7–35.0), and 51.4% in Colombia (38/74; exact 95% CI 39.4–63.2). The migalastat-amenable variants that were reported differed by country, with little overlap among the cohorts in Brazil, Argentina, and Colombia in the most common variants (Supplementary Table S1). In the cohort of individuals with amenable variants in Brazil, the most common migalastat-amenable variants reported were D313Y (present in 26.8% of individuals with amenable variants), R118C (26.5%), and R356W (13.5%). In the cohort of individuals with amenable variants in Argentina, the most common amenable variants reported were D264Y (31.3%), C174G (21.9%), and T194I (15.6%). In the cohort of individuals with amenable variants in Colombia, the most common amenable variants reported were G80D (71.1%), P214L (13.2%), and D182G (10.5%).

### 3.2 Demographic characteristics of individuals with migalastat-amenable variants

Median age (in years) at the study reference date (July 1, 2021) for individuals with migalastat-amenable variants was 45.6 in Brazil, 44.1 in Argentina, and 21.8 in Colombia (Table 1). Overall, there were more females than males in the study population of individuals with migalastat-amenable variants (Table 1), as would be expected based on Mendelian inheritance of an X-linked gene.

**TABLE 1:** Demographics of patients with migalastat-amenable variants by country and overall.

Demographics	Brazil (n = 650)	Argentina (n = 32)	Colombia (n = 38)	Total (N = 720)
Age, years*				
Mean (SD)	46.1 (20.3)	41.8 (17.3)	28.6 (18.9)	45.0 (20.5)
Median (Q1–Q3)	45.6 (31.6–61.1)	44.1 (25.9–57.2)	21.8 (16.4–44.3)	45.0 (30.4–60.8)
Sex, *n* (%)				
Male	265 (40.8)	15 (46.9)	7 (18.4)	287 (39.9)
Female	383 (58.9)	15 (46.9)	30 (78.9)	428 (59.4)
Missing	2 (0.3)	2 (6.3)	1 (2.6)	5 (0.7)
Ethnicity, *n* (%)				
African American	1 (0.2)	0	0	1 (0.1)
Asian	0 (0.0)	0	0	0 (0.0)
Hispanic	0 (0.0)	28 (87.5)	0	28 (3.9)
Mestizo	0 (0.0)	0	38 (100)	38 (5.3)
White	3 (0.5)	2 (6.3)	0	5 (0.7)
Other	646 (99.4)	2 (6.3)	0	648 (90.0)
Missing	0	0	0	0

* Determined in reference to July 1, 2021. Q1–Q3, first quartile–third quartile (interquartile range); SD, standard deviation.

### 3.3 Clinical characteristics of individuals with migalastat-amenable variants

Owing to limited data collection in Brazil, information about clinical characteristics was available only for individuals in Argentina and Colombia (Tables 2, 3). For those with available data, the median eGFR in individuals with migalastat-amenable variants aged ≥18 years was 95 mL/min/1.73 m^2^ (interquartile range [IQR], 71–111; n = 26) in Argentina and, although data were limited, 84 mL/min/1.73 m^2^ (IQR, 53–122; n = 9) in Colombia. Approximately half of those aged ≥18 years with eGFR data in both countries had some degree of renal impairment (Table 2). In Argentina, median left ventricular mass index (LVMi) in individuals with amenable variants aged ≥18 years was 86.5 g/m^2^ (IQR, 71.0–107.0; n = 14) in females and 119.0 g/m^2^ (IQR, 103.0–135.0; n = 13) in males (Table 2). Data for LVMi in Colombia were very limited (Table 2). Clinical evidence of Fabry disease was recorded in individuals with migalastat-amenable variants in both Argentina and Colombia, including the classic Fabry disease signs and symptoms of acroparesthesia, abdominal pain, angiokeratoma, and cornea verticillata (Table 3).

**TABLE 2:** Renal and cardiac clinical characteristics in individuals with Fabry disease and migalastat-amenable variants.

Clinical characteristics	Argentina (n = 32)	Colombia (n = 38)
eGFR in adults, mL/min/1.73 m^2^ *		
*n*	26	9
Mean (SD)	89.6 (37.2)	118.6 (129.9)
Median (Q1–Q3)	95.0 (71.0–111.0)	84.0 (53.0–122.0)
Renal impairment category in adults, n (%)*	​	​
Normal (eGFR ≥90 mL/min/1.73 m^2^)	14 (53.8)	4 (44.4)
Mild (eGFR ≥60–<90 mL/min/1.73 m^2^)	8 (30.8)	2 (22.2)
Moderate (eGFR ≥30–<60 mL/min/1.73 m^2^)	1 (3.8)	2 (22.2)
Severe (eGFR <30 mL/min/1.73 m^2^)	3 (11.5)	1 (11.1)
Albumin/creatinine ratio, mg/g^†^		
*n*	3	19
Mean (SD)	7.3 (0.8)	2.2 (2.5)
Median (Q1–Q3)	7.30 (6.50–8.00)	1.64 (0.00–4.18)
Proteinuria, mg/24 h		
*n*	32	27
Mean (SD)	223.7 (324.1)	100.8 (97.5)
Median (Q1–Q3)	92.0 (47.0–260.0)	90.0 (45.0–134.0)
Proteinuria category, n (%)^‡^	​	​
Normal (<300 mg/24 h)	26 (81.3)	26 (68.4)
Mild (300–1,000 mg/24 h)	5 (15.6)	1 (2.6)
Severe (>1,000 mg/24 h)	1 (3.1)	0
Missing	0	11 (28.9)
LVMi in adults, g/m^2^ ^§^		
Female	​	​
*n*	14^‖^	3
Mean (SD)	87.6 (26.6)	56.3 (17.0)
Median (Q1–Q3)	86.5 (71.0–107.0)	57.0 (39.0–73.0)
Male	​	​
*n*	13^‖^	2
Mean (SD)	132.6 (68.0)	36.5 (6.4)
Median (Q1–Q3)	119.0 (103.0–135.0)	36.5 (32.0–41.0)
Posterior wall thickness in adults, mm^§^		
*n*	29	11
Mean (SD)	9.5 (2.7)	6.2 (3.9)
Median (Q1–Q3)	9.0 (8.0–11.0)	8.0 (1.0–9.0)

* Data included if the date on which renal function was assessed was documented and patient was aged ≥18 years on that date. Renal impairment category percentages are based on the total number of individuals with eGFR, data in each country.

^†^ Where applicable, albumin/creatinine ratio was converted from mg/mmol to mg/g using the following equation: mg/mmol value/0.113.

^‡^ Proteinuria category percentages are based on the total number of individuals in the study population in each country.

^§^ Data included if the date on which the echocardiogram was performed was documented and patient was aged ≥18 years on that date. LVMi, is reported by sex due to the significant difference in normative cardiac size between females and males (St Pierre et al., 2022).

^‖^ Data for two adults were excluded as sex could not be confirmed using the data extracted from their medical records. eGFR, estimated glomerular filtration rate; LVMi, left ventricular mass index; Q1–Q3, first quartile–third quartile (interquartile range); SD, standard deviation.

**TABLE 3:** Signs and symptoms of Fabry disease in individuals with migalastat-amenable variants.

Signs and symptoms	Patients, *n* (%)	
	Argentina (n = 32)	Colombia (n = 38)
Classic Fabry disease signs and symptoms		
Acroparesthesia	18 (56.3)	23* (60.5)
Abdominal pain	9 (28.1)	14* (36.8)
Angiokeratoma	10 (31.3)	11* (28.9)
Cornea verticillata	11 (34.4)	1* (2.6)
Renal involvement		
Gb3 inclusions on renal biopsy	2* (6.3)	4* (10.5)
Dialysis	3* (9.4)	0*
Renal transplantation	1 (3.1)	0*
Cardiac signs and symptoms		
LVH in adults^†^	13^‡^ (40.6)	0^§^
Arrhythmias	2 (6.3)	6* (15.8)
Coronary disease	2 (6.3)	5* (13.2)
Neurological involvement		
Depression symptoms	1 (3.1)	15* (39.5)
Cerebrovascular involvement	8 (25.0)	4* (10.5)
Cerebrovascular – ischemic	8 (25.0)	4 (10.5)
Cerebrovascular – hemorrhagic	0	0
TIA	0*	3* (7.9)
Stroke	0*	0*
Gastrointestinal symptoms		
Indigestion	6 (18.8)	10* (26.3)
Diarrhea	7 (21.9)	6* (15.8)
Reflux	1 (3.1)	9* (23.7)
Constipation	2 (6.3)	1* (2.6)

* Data are missing for three patients for the values relating to arrhythmias, coronary disease, depression symptoms and constipation, for one patient for the values relating to Gb3 inclusions (Colombia), dialysis (both countries) and renal transplantation, and for two patients for all other values.

^†^ Defined as an LVMi >95 g/m^2^ in women and >115 g/m^2^ in men in patients ≥18 years of age on the date on which the echocardiogram was performed.

^‡^ Out of 29 adults with data for LVMi (although it was not possible to confirm the sex of 2/29 using the data extracted from their medical records, it was possible to determine whether they had LVH, because one had LVMi ≤95 g/m^2^ and the other had LVMi >115 g/m^2^); percentage relates to the overall population (n = 32).

^§^ Out of five adults with data for LVMi and sex. Gb3, globotriaosylceramide; LVH, left ventricular hypertrophy; LVMi, left ventricular mass index; TIA, transitory ischemic attack.

## 4 Discussion

In this retrospective study conducted at centers in Brazil, Argentina, and Colombia, the proportion of individuals diagnosed with Fabry disease who had at least one migalastat-amenable *GLA* variant varied by country. These differences should be interpreted in the context of local diagnostic and referral pathways, cohort composition, and differences in healthcare professional interpretation of the pathogenicity of variants of uncertain significance (VUS), which may have influenced the reported amenability estimates. The most frequently reported migalastat-amenable variants differed substantially between countries, with D313Y, R118C, and R356W most common in Brazil; D264Y, C174G, and T194I in Argentina; and G80D, P214L, and D182G in Colombia. This limited overlap suggests that the prevalence estimates are not directly comparable between countries and may reflect, at least in part, the variants most frequently encountered in each participating center’s population.

Interpretation is further complicated by the inclusion of variants for which clinical significance, including pathogenicity, remains uncertain or continues to evolve. Migalastat amenability is a classification based on whether a variant meets criteria for amenability (Benjamin et al., 2017); it does not, by itself, establish pathogenicity, clinical severity, or expected clinical response in an individual patient. In Brazil D313Y and R118C accounted for more than 50% of the migalastat-amenable population, while in Colombia G80D accounted for approximately 70% (Supplementary Table S1). As a result, differences in the interpretation of these variants could have a substantial effect on the estimated prevalence of migalastat-amenable variants in these cohorts.

The cohorts also differed in their sources of ascertainment, which may have influenced both the apparent prevalence of migalastat-amenable variants and the observed clinical characteristics. In Brazil, index patients were identified from dialysis clinics and additional related individuals through family cascade screening, whereas the Argentina and Colombia cohorts comprised patients from principal investigators’ and satellite clinics. These different recruitment pathways could have enriched the cohorts for different clinical profiles, including individuals with renal involvement or other end-organ damage, and relatives identified through cascade screening. Although the available clinical data suggest that the cohorts may have included individuals with differing clinical profiles, the absence of standardized phenotype classification and variability among physicians in distinguishing classic from later-onset Fabry disease limits a robust assessment of whether each country cohort primarily reflected classic Fabry disease, later-onset disease, or mixed phenotypes.

The presentation, clinical significance, and management of VUS in Fabry disease is an evolving discussion (Germain et al., 2022; Gragnaniello et al., 2023). For example, a recently published study reported data from patients with Fabry disease identified via a screening program for people undergoing hemodialysis in Brazil (Paraiba Valley and Eastern São Paulo) and potentially affected relatives (N = 82). There were group-level differences in globotriaosylsphingosine (lyso-Gb3) levels, and α-Gal A activity in patients considered by the investigators to have pathogenic variants versus those considered to have VUS (Paz et al., 2023). However, on considering data for individual patients, there was some overlap in lyso-Gb3 levels and all had α-Gal A activity <15.3 mmol/L/h (Paz et al., 2023), supporting the notion that neither one of these measures on its own can be used to make a clinical decision. Biopsied tissue-guided inclusion identification might be of diagnostic value in such situations. In the PRIME study, the identification of Brazilian index patients through hemodialysis clinics suggests that renal disease was an important route of ascertainment in this cohort. However, because detailed clinical data were not available for most individuals in Brazil, the extent and cause of renal involvement could not be robustly characterized. Further research is required to characterize the phenotypic and genomic traits of VUS to elucidate their variability and potential impact, for example, using renal biopsy to identify Gb3 inclusions and taking detailed family histories and pedigrees to ascertain segregation of disease characteristics with the variant in question. More broadly, computational and systems-level approaches, including disease-gene prediction and tissue-specific gene coexpression network analyses, may help prioritize candidate disease-related genes and biological contexts for further investigation (He et al., 2020; Xiang et al., 2022). In Fabry disease, however, clinical interpretation of *GLA* variants still requires integration of genetic findings with biochemical, phenotypic, histopathological and family segregation data. Furthermore, variant classifications should be checked regularly in large databases as new information is added. This underscores the need for individualized disease burden assessment to support clinical decision-making.

Despite the presence of VUS and younger patients in the Colombia group, patients in the Argentina and Colombia groups appeared to have both subjective and objective manifestations of Fabry disease. A recent cohort study of 17 adult patients with Fabry disease and migalastat-amenable variants in Argentina (with a mean age for males and females of 45.8 and 49.9 years, respectively), assessed prior to treatment with migalastat, also found evidence of Fabry disease signs and symptoms and renal impairment in this population (Jaurretche et al., 2024).

This study has several strengths. To our knowledge, PRIME is the first study to report the prevalence of migalastat-amenable *GLA* variants across multiple Latin American countries, and includes a large overall cohort for a rare disease population. The inclusion of centers from Brazil, Argentina, and Colombia provides insight into migalastat amenability across different clinical settings, referral pathways and variant spectra.

These strengths should be considered alongside several limitations when evaluating the findings. Data were collected retrospectively from medical records at centers in three countries and reflected real-world local approaches to Fabry disease diagnosis, referral, clinical assessment, and variant interpretation rather than a standardized cross-country diagnostic protocol*.* In most centers in Argentina, for example, variant pathogenicity is based on factors such as reduced α-Gal A activity, variant type, increased levels of lyso-Gb3, presence of Gb3 inclusions in specific cell types and the age at which typical symptoms or serious events first occur (Politei et al., 2025). In Columbia, when a VUS or new variant is detected, an assessment of patient symptomology, lyso-Gb3 levels and enzymatic activity is carried out, as well as reviewing the variant according to the American College of Medical Genetics and Genomics/Association for Molecular Pathology classification criteria (Harrison et al., 2019); similarly in Brazil, diagnosis confirmation requires investigation of phenotype, biomarkers and even use of *in silico* prediction tools (Silva et al., 2022). Any differences in diagnosis and recruitment pathways may introduce ascertainment bias and limit direct comparison of amenability estimates between countries. The study was not designed to evaluate founder effects or regional genetic ancestry, as ancestry data and detailed pedigree information were not systematically captured. In addition, the cohorts were also recruited from a limited number of participating centers and may reflect clustering of familial variants within those centers rather than country-wide variant distributions. The ethnicity of nearly all individuals in Brazil was categorized as “Other” (i.e., did not fit within a named category), reflecting the heterogeneity of the population and limiting interpretation of variant impact by ethnicity or ancestry. Further characterization of variant impact in ethnically diverse populations is an important direction for future study. Disease characteristics were recorded only for the smaller cohorts in Argentina and Colombia, and data for some assessments (e.g., LVMi) were sparse, which may reflect local practices in disease management or the complications with which patients presented. As a result, genotype-phenotype interpretation is limited, and the clinical findings should be considered descriptive and hypothesis-generating.

In conclusion, variability was found in the prevalence of migalastat-amenable variants in the Fabry disease population at centers in Brazil, Argentina, and Colombia, with local approaches to the identification of Fabry disease and differing approaches to interpreting VUS likely contributing to the variability. In those individuals for whom data were available, there appeared to be both subjective and objective manifestations of Fabry disease.

## Data Availability

The data analyzed in this study is subject to the following licenses/restrictions: The de-identified individual participant data that underlie the results reported in this article (including text, tables, figures, and Supplementary Material) will be made available together with the research protocol and data dictionaries, for non-commercial, academic purposes. Additional supporting documents may be available upon request. Investigators will be able to request access to these data and supporting documents via a data sharing portal (www.biomarin.com/our-science/funding-and-support/publication-data-request) beginning 6 months and ending 2 years after publication. Data associated with any ongoing development program will be made available within six (6) months after approval of relevant product. Requests must include a research proposal clarifying how the data will be used, including proposed analysis methodology. Research proposals will be evaluated relative to publicly available criteria available at www.biomarin.com/our-science/funding-and-support/publication-data-request to determine if access will be given, contingent upon execution of a data access agreement with BioMarin Pharmaceutical Inc. Requests to access these datasets should be directed to www.biomarin.com/our-science/funding-and-support/publication-data-request.
