# Supplementary material for: Prevalence of migalastat-amenable variants in patients with Fabry disease from Brazil, Argentina, and Colombia: an observational study
Source: Front Genet. 2026 Oct 5;17:1876053. doi: 10.3389/fgene.2026.1876053 (PMC13678124; doi:10.3389/fgene.2026.1876053)
Supplement: Supplementary file 1 [file Table1.docx]

# Supplementary information

Supplement to: Politei JM *et al*. Prevalence of migalastat-amenable variants in patients with Fabry disease from Brazil, Argentina, and Colombia: an observational study

**Supplementary Table 1.** Migalastat-amenable variants identified in the study population

| **Amenable α-gal A variant** | **n (%) of patients with at least one migalastat-amenable variant*** | | | |
| --- | --- | --- | --- | --- |
|  | **Brazil (n = 650)** | **Argentina (n = 32)** | **Colombia (n = 38)** | **Total (N = 720)** |
| D313Y | 174 (26.8) | 0 | 0 | 174 (24.2) |
| R118C | 172 (26.5) | 0 | 0 | 172 (23.9) |
| R356W | 88 (13.5) | 0 | 0 | 88 (12.2) |
| V269M | 53 (8.2) | 0 | 0 | 53 (7.4) |
| M290I | 40 (6.2) | 0 | 0 | 40 (5.6) |
| G80D | 0 | 0 | 27 (71.1) | 27 (3.8) |
| N215S | 16 (2.5) | 2 (6.3) | 0 | 18 (2.5) |
| A143T | 11 (1.7) | 0 | 0 | 11 (1.5) |
| D264Y | 0 | 10 (31.3) | 0 | 10 (1.4) |
| W349S | 10 (1.5) | 0 | 0 | 10 (1.4) |
| N228S | 9 (1.4) | 0 | 0 | 9 (1.3) |
| A368T | 8 (1.2) | 0 | 0 | 8 (1.1) |
| P323T | 8 (1.2) | 0 | 0 | 8 (1.1) |
| C174G | 0 | 7 (21.9) | 0 | 7 (1.0) |
| M187T | 7 (1.1) | 0 | 0 | 7 (1.0) |
| R363H | 6 (0.9) | 1 (3.1) | 0 | 7 (1.0) |
| L3P | 5 (0.8) | 0 | 0 | 5 (0.7) |
| P214L | 0 | 0 | 5 (13.2) | 5 (0.7) |
| T194I | 0 | 5 (15.6) | 0 | 5 (0.7) |
| D182G | 0 | 0 | 4 (10.5) | 4 (0.6) |
| F69L | 4 (0.6) | 0 | 0 | 4 (0.6) |
| G85D | 0 | 4 (12.5) | 0 | 4 (0.6) |
| N34D | 4 (0.6) | 0 | 0 | 4 (0.6) |
| R112H | 4 (0.6) | 0 | 0 | 4 (0.6) |
| V269A | 4 (0.6) | 0 | 0 | 4 (0.6) |
| I319T | 3 (0.5) | 0 | 0 | 3 (0.4) |
| M284T | 3 (0.5) | 0 | 0 | 3 (0.4) |
| R220Q | 3 (0.5) | 0 | 0 | 3 (0.4) |
| R301G | 3 (0.5) | 0 | 0 | 3 (0.4) |
| D109G | 0 | 2 (6.3) | 0 | 2 (0.3) |
| D83N | 2 (0.3) | 0 | 0 | 2 (0.3) |
| G35V | 2 (0.3) | 0 | 0 | 2 (0.3) |
| L189F | 2 (0.3) | 0 | 0 | 2 (0.3) |
| A15G | 1 (0.2) | 0 | 0 | 1 (0.1) |
| F113L | 1 (0.2) | 0 | 0 | 1 (0.1) |
| G258V | 0 | 1 (3.1) | 0 | 1 (0.1) |
| G360A | 0 | 0 | 1 (2.6) | 1 (0.1) |
| G373S | 1 (0.2) | 0 | 0 | 1 (0.1) |
| G375A | 0 | 0 | 1 (2.6) | 1 (0.1) |
| I198T | 1 (0.2) | 0 | 0 | 1 (0.1) |
| K140T | 1 (0.2) | 0 | 0 | 1 (0.1) |
| M42I | 1 (0.2) | 0 | 0 | 1 (0.1) |
| R356Q | 1 (0.2) | 0 | 0 | 1 (0.1) |
| T282I | 1 (0.2) | 0 | 0 | 1 (0.1) |
| Y152D | 1 (0.2) | 0 | 0 | 1 (0.1) |

*Population includes all individuals diagnosed with Fabry disease (based on genetic testing and/or α-gal A enzymatic activity) who had a variant amenable to migalastat. α-gal A, α-galactosidase A.
